# Cellular and molecular basis of haploidentical hematopoietic stem cell transplantation in the successful treatment of high-risk leukemias: role of alloreactive NK cells

**DOI:** 10.3389/fimmu.2013.00015

**Published:** 2013-02-01

**Authors:** Franco Locatelli, Daniela Pende, Maria C. Mingari, Alice Bertaina, Michela Falco, Alessandro Moretta, Lorenzo Moretta

**Affiliations:** ^1^Department of Pediatric Hematology/Oncology, Istituto Di Ricovero e Cura a Carattere Scientifico, Ospedale Pediatrico Bambino GesùRome, Italy; ^2^Università di PaviaPavia, Italy; ^3^Istituto Di Ricovero e Cura a Carattere Scientifico, Azienda Ospedaliera Universitaria San Martino – Istituto Nazionale per la Ricerca sul CancroGenoa, Italy; ^4^Department of Experimental Medicine, Centre of Excellence for Biomedical Research, University of GenoaGenoa, Italy; ^5^Istituto Di Ricovero e Cura a Carattere Scientifico, Istituto Giannina GasliniGenoa, Italy

**Keywords:** natural killer cells, killer Ig-like receptors, NK alloreactivity, acute myeloid leukemia, acute lymphoblastic leukemia, haploidentical hemopoietic stem cell transplantation, graft-vs-host disease

## Abstract

Natural killer (NK) cells are involved in innate immune responses and play a major role in tumor surveillance and in defense against viruses. Human NK cells recognize human leukocyte antigen (HLA) class I molecules via surface receptors [killer immunoglobulin-like receptor (KIR) and NKG2A] delivering signals that inhibit NK cell function and kill HLA class I-deficient target cells, a frequent event in tumors or virus-infected cells. NK cell triggering is mediated by activating receptors that recognize ligands expressed primarily on tumors or virus-infected cells. NK cells play also a key role in the cure of high-risk leukemias. Thus, donor-derived “alloreactive” NK cells are fundamental effectors in adult acute myeloid leukemia and in pediatric acute lymphoblastic leukemia patients undergoing haploidentical hematopoietic stem cell transplantation (HSCT). Alloreactive NK cells mediate killing of leukemia cells and patient’s dendritic cell, thus preventing respectively leukemic relapses and graft-vs-host responses. Cytofluorimetric analysis of KIRs expressed by NK cells allows to define the size of the alloreactive NK subset and the selection of the best potential donor. Recently, it has been shown that also the expression of activating KIRs, in particular the (C2-specific) KIR2DS1, may contribute to donor NK alloreactivity. It has also been established a correlation between the size of the alloreactive NK cell population and the clinical outcome. Notably, the alloreactive NK cells derived from donor’s hematopoietic stem cells are generated and persist in patients over time. The high survival rates of patients undergoing haploidentical HSCT highlight an important new reality in the setting of allograft performed to cure otherwise fatal leukemias. Novel approaches are in progress to further improve the clinical outcome based on the infusion of donor alloreactive NK cells either as a component of the transplanted cell population or as *in vitro* expanded NK cells.

## HAPLOIDENTICAL HEMOPOIETIC STEM CELL TRANSPLANTATION

For over 40 years, allogeneic hematopoietic stem cell transplantation (allo-HSCT) from an human leukocyte antigen (HLA)-matched donor, either related or unrelated, has been increasingly used to treat patients affected by several malignant or non-malignant disorders. Thanks to this procedure, thousands of subjects have been cured of their original disease (Copelan, 2006). However, only 25% of patients who need an allograft have an HLA-identical sibling available and for <60% of the remaining patients a suitable, HLA-compatible, unrelated volunteer can be found (Rocha and Locatelli, 2008). In the absence of an HLA-matched donor, alternative donors/sources of hematopoietic stem cells (HSC), such as unrelated umbilical cord blood (UCB) and HLA-haploidentical relatives, are being increasingly used (Gluckman, 2006; Rocha and Locatelli, 2008; Locatelli et al., 2009). In particular, the majority of patients have a family member, identical for one HLA-haplotype and fully mismatched for the other (i.e., haploidentical), who can immediately serve as HSC donor (Martelli et al., 2002; Locatelli et al., 2009). Thus, HSCT from an HLA-haploidentical relative (haplo-HSCT) offers an immediate transplant treatment *virtually* to *any* patients lacking a matched donor or a suitable UCB unit.

A major breakthrough in the history of successful haplo-HSCT was the demonstration that an efficient T cell-depletion of the graft prevented both acute and chronic graft-vs-host disease (GvHD), even when the donor was a relative differing for an entire HLA-haplotype from the recipient (Reisner et al., 1983). The importance of T cell-depleted haplo-HSCT was first shown in children with severe combined immunodeficiency (SCID; Reisner et al., 1983) and it can now be estimated that hundreds of SCID patients have been transplanted worldwide using an HLA-haploidentical related donor, with a high rate of long-term, either partial or complete, immune reconstitution (Antoine et al., 2003). However, while the infusion of bone marrow (BM) cells obtained from an HLA-haploidentical relative was associated with a high engraftment rate in children with SCID, it was associated with an unacceptably high incidence of graft failure in patients with acute leukemia (Reisner and Martelli, 1999). In these cases, due to the extensive T cell-depletion of the graft, the balance between competing host and donor T cells shifts in favor of the unopposed host-vs-graft rejection (Reisner and Martelli, 1999). As a possible solution to this obstacle, the use of “megadoses” of granulocyte colony-stimulating factor (G-CSF)-mobilized peripheral blood-derived HSC was shown, in animal models, to overcome the barrier of HLA incompatibility and to elude the residual anti-donor T lymphocyte reactivity of the recipient (Bachar-Lustig et al., 1995). An effective translation of this approach into the clinical setting was first reported in a pilot study performed in adults with acute leukemia (Aversa et al., 1994). In this study, Aversa et al. (1994) transplanted “megadoses” of T cell-depleted HSC from BM or G-CSF-mobilized peripheral blood without any subsequent pharmacological GvHD prophylaxis. The reported engraftment rate was above 90% with a cumulative incidence of both grade II–IV acute and chronic GvHD below 10%. Clinical trials performed using purified CD34^+^ cells have confirmed that sustained engraftment of donor hematopoiesis, without the occurrence of GvHD, can be obtained in the majority of adult patients and that a substantial proportion of them, especially when affected by acute myeloid leukemia (AML) or myelodysplastic syndromes, become long-term survivors (Aversa et al., 1998; Ruggeri et al., 2002).

In view of the role played by donor T cells in mediating the graft-vs-leukemia (GvL) effect, it could be expected that a relevant proportion of patients given this type of allograft would experience leukemia relapses. This expectation was only partly confirmed by clinical results, since among adult patients affected by AML, a subgroup of patients given T cell-depleted HSCT from an HLA-disparate relative had a particularly low risk of leukemia relapse (Aversa et al., 1998; Ruggeri et al., 2002). These patients were transplanted from a donor having natural killer (NK) cells that were “alloreactive” toward recipient targets. NK cell alloreactivity was originally described by Moretta et al. (1990a) over 20 years ago when killing of allogeneic lymphoblasts was observed *in vitro* and associated with defined NK cell subsets (Moretta et al., 1990a) identified by the expression or lack thereof of novel surface molecules (Moretta et al., 1990b), subsequently identified as HLA class I-specific receptors (Ciccone et al., 1992b, 1994; Moretta et al., 1993, 1996; Wagtmann et al., 1995). The emergence of the concept of the efficacy of NK cell alloreactivity in this transplantation setting has represented a sort of revolution in the field of haplo-HSCT, underlining for the first time that not only adaptive immunity, but also innate immunity is a crucial element for guaranteeing a successful clinical outcome (Moretta et al., 2008; Locatelli et al., 2009). Indeed, it became evident that the therapeutic effect of haplo-HSCT is largely dependent on the GvL effect exerted by NK cells which originate from donor HSC (Ruggeri et al., 2002; Moretta et al., 2008; Locatelli et al., 2009) and largely contribute to eradicate leukemia cells surviving the preparative regimen.

Thus, while for many years the absence of the T cell-mediated GvL effect was considered to render the recipients of a T cell-depleted allograft more susceptible to leukemia relapse (Horowitz et al., 1990), it is now evident that, in haplo-HSCT, an efficient GvL effect can be mediated by donor-derived alloreactive NK cells which compensate for the lack of T cell intervention.

## NK CELL RECEPTORS AND FUNCTION

Natural killer cells are important players of the innate immunity. They are regulated by a number of receptors that finely tune potent effector functions, including cytolytic activity against different target cells and release of cytokines that play a major role in inflammation and immunoregulation (Trinchieri, 1989; Moretta et al., 1994; Janeway and Medzhitov, 2002; Moretta and Moretta, 2004).

A group of inhibitory receptors interact specifically with major histocompatibility (MHC) class I molecules (Ciccone et al., 1992b; Moretta et al., 1993, 1996; Long, 1999). These receptors prevent NK cell-mediated attack against normal (i.e., MHC class I^+^) autologous cells. Cells in which MHC class I expression is compromised/downregulated (e.g., by tumor transformation or viral infection) become susceptible to NK-mediated killing. In humans, the inhibitory receptors for HLA class I molecules, namely: (1) killer immunoglobulin (Ig)-like receptors (KIR2DL/3DL) that belong to the Ig superfamily and are specific for determinants shared by groups of HLA-A, -B, or -C allotypes (referred to as KIR-ligands; reviewed in Moretta et al., 1996; Lanier, 1998; Long, 1999; **Table 1**), (2) CD94/NKG2A, a heterodimer related to C-type lectins that recognizes HLA-E, an HLA class Ib molecule (Lanier, 1998; Lopez-Botet et al., 2000), and (3) LILRB1 (ILT2, LIR-1, CD85j) that displays broad HLA class I specificity and interacts with UL18 human cytomegalovirus (HCMV) glycoprotein (Colonna et al., 1997; Cosman et al., 1997). Notably, activating forms of KIRs (KIR2DS/3DS; Moretta et al., 1995, 1996; Lanier, 1998), and CD94/NKG2C also exist. Activating KIRs may be relevant for recognition and killing of leukemia cells and dendritic cells (DCs; see below), while CD94/NKG2C appears to be involved in the control of HCMV infections (Gumà et al., 2004; Della Chiesa et al., 2012; Foley et al., 2012). In addition, NK cells are equipped with several triggering receptors responsible for NK cell activation in the process of natural cytotoxicity. An important role in tumor cell killing is exerted by NKp46 (Sivori et al., 1997; Pessino et al., 1998), NKp30 (Pende et al., 1999), and NKp44 (Vitale et al., 1998; Cantoni et al., 1999), a group of activating receptors that are mostly restricted to NK cells and that are collectively named “natural cytotoxicity receptors” (NCRs). In particular, NKp46 expressed both in human and in mouse NK cells represents the most reliable marker for NK cell identification (Sivori et al., 1997; Walzer et al., 2007). The cellular ligands recognized by these receptors are still elusive, with the exception of B7-H6, a ligand for NKp30 (Brandt et al., 2009). Another receptor that plays a major role in NK cell-mediated recognition and killing of some tumors is NKG2D, a type II membrane protein characterized by a lectin-like domain (Wu et al., 1999). NKG2D recognizes the stress-inducible MHC class I-related chain A/B (MICA/B) or UL16-binding proteins (ULBP; Raulet, 2003). Other activating receptors include 2B4 (Moretta et al., 1992; Valiante and Trinchieri, 1993) specific for CD48, NK, T, and B cell antigen (NTB-A; Bottino et al., 2001) mediating homotypic interactions, NKp80 (Vitale et al., 2001) specific for AICL1 (Welte et al., 2006), DNAM-1 (Shibuya et al., 1996) specific for poliovirus receptor (PVR, CD155), and Nectin-2 (CD112; Bottino et al., 2003) also involved in cell-to-cell adhesion and in leukocyte extravasation (Reymond et al., 2004). Notably, PVR and Nectin-2 are frequently over-expressed on tumor cells and leukemia blasts (Bottino et al., 2003). Recognition of self-ligands that are induced by viral infection, tumor transformation, and in general cell stress may represent an important mechanism by which NK cells can identify and remove abnormal cells.

**Table 1:** KIRs and KIR-ligands.

KIR	Domain composition	KIR-ligand	Function	Reference
2DL1	D1 + D2	HLA-C^Lys80^ (C2)	Inhibitory	Ciccone et al. (1992a), Biassoni et al. (1995)
2DL2/2DL3	D1 + D2	HLA-C^Asn80^ (C1), HLA-B*46:01, HLA-B*73:01 Low affinity: HLA-C^Lys80^ (C2)	Inhibitory	Ciccone et al. (1992a), Biassoni et al. (1995), Moesta et al. (2008)
2DL4	D0 + D2	HLA-G	Inhibitory and activating*	Rajagopalan et al. (2001)
2DL5	D0 + D2	Unknown	Inhibitory	
3DL1	D0 + D1 + D2	HLA-B^Bw4^ and some HLA-A^Bw4^	Inhibitory	Gumperz et al. (1997), Stern et al. (2008)
3DL2	D0 + D1 + D2	HLA-A*03 and HLA-A*11	Inhibitory	Döhring et al. (1996), Pende et al. (1996)
2DS1	D1 + D2	HLA-C^Lys80^ (C2)	Activating	Stewart et al. (2005), Chewning et al. (2007)
2DS2	D1 + D2	Unknown	Activating	
2DS3	D1 + D2	Unknown	Activating	
2DS4	D1 + D2	HLA-A*11 and some HLA-C alleles	Activating	Graef et al. (2009)
2DS5	D1 + D2	Unknown	Activating	
3DS1	D0 + D1 + D2	HLA-B^Bw4^ (?)	Activating	Martin et al. (2002)

* *KIR2DL4 may function as an inhibitory receptor in cytotoxicity while it triggers IFN-γ production*.

## KIR REPERTOIRE AND SPECIFICITY FOR HLA CLASS I ALLELES

The ability of NK cells to sense allelic differences on hematopoietic target cells was first suggested by the hybrid resistance phenomenon in which NK cells can reject parental BM grafts in F1 hybrid mice (Bennet, 1987). Studies in both humans and mice clarified the general mechanisms underlying NK cell function and their capability of selectively killing tumor cells. In humans, two surface molecules expressed by subsets of NK cells that were capable of modulating NK cell function were identified (Moretta et al., 1990a,b, 1993; Wagtmann et al., 1995). They were shown to function as inhibitory receptors specific for distinct HLA-C alleles (Moretta et al., 1993). Molecular cloning revealed novel members of the Ig superfamily characterized by two extracellular Ig-like domains (KIR2D) and by a cytoplasmic tail containing two immunoreceptor tyrosine-based inhibition motif (ITIM; Moretta et al., 1990a,b, 1993; Wagtmann et al., 1995). Three Ig-like domain KIRs (KIR3D) were also identified (Colonna and Samaridis, 1995). They recognize either a group of HLA-B alleles sharing the HLA-Bw4 supertypic specificity or certain HLA-A alleles.

Among the activating forms of KIRs, the specificity for HLA class I molecules has been unequivocally documented only for KIR2DS1 and KIR2DS4 (**Table 1**; Moretta et al., 1995; Stewart et al., 2005; Chewning et al., 2007; Graef et al., 2009). KIRs are clonally distributed on NK cells and individual cells express different sets of inhibitory or activating KIRs. Notably, most (but not all) NK cells express at least one self-reacting inhibitory receptor, either a KIR or CD94/NKG2A (Moretta et al., 1996).

While in an autologous setting NK cells can kill only cells that do not express sufficient HLA class I molecules (Ciccone et al., 1994), in a non-self environment NK cells may kill allogeneic cells. It became evident that such “alloreactive” NK cells could kill allogeneic cells, both *in vitro* and *in vivo*, when they expressed inhibitory KIRs that did not recognize HLA class I alleles on target cells (Ciccone et al., 1992b, 1994; Moretta et al., 1993; Pende et al., 2005). In addition, these alloreactive NK cells should not express CD94/NKG2A^+^ (Pende et al., 2005) because HLA-E molecules are present in all HLA class I^+^ cells.

Notably, other factors may greatly contribute to NK alloreactivity. In particular, killing of target cells may also depend on the surface density of certain activating receptors (such as NCRs) on NK cells and on the expression of their ligands on target cells (Costello et al., 2002; Pende et al., 2005). More importantly, activating KIRs (in particular KIR2DS1) were shown to play a substantial role in mediating alloreactivity (Chewning et al., 2007; Pende et al., 2009). KIR2DS1 activating receptor recognizes the C2 specificity (Chewning et al., 2007). It is worthy to note that, in NK cells derived from C1/C2 or C1/C1 donors, activation via KIR2DS1 may overcome also the KIR2DL2/3-mediated inhibition, resulting in an efficient lysis of C2/C2 leukemic cells (Pende et al., 2009). In addition, KIR2DS1 can overcome the CD94/NKG2A-mediated inhibition, again resulting in killing of C2/C2 leukemias. Thus, the expression of KIR2DS1 may reveal NK cells endowed with potent alloreactivity and allow a more precise definition of the size of the alloreactive NK cell subset (Pende et al., 2009).

## IDENTIFICATION OF ALLOREACTIVE NK CELLS

Phenotypic identification of the alloreactive NK cell subset and assessment of the NK cytolytic activity against leukemic cells represent important criteria in donor selection. Multi-color flow-cytometric analysis using appropriate combinations of monoclonal antibodies (mAb) allows the identification and definition of the size of the alloreactive NK cell population (Chewning et al., 2007; Pende et al., 2009). Substantial progress has been made recently after the identification of mAbs discriminating between inhibitory and activating KIRs. Thanks to these mAbs, it is now possible to distinguish KIR3DL1 from KIR3DS1, KIR2DL1 from KIR2DS1, and KIR2DL3 (but not KIR2DL2) from KIR2DS2 (Pende et al., 2009). This is most important because the expression of activating KIRs, in particular KIR2DS1, recognizing alleles belonging to the C2 specificity may exert a positive effect and greatly contribute to NK alloreactivity, provided that patient’s cells express C2 alleles. Notably, the beneficial effect is more evident in leukemia blasts of pediatric acute lymphoblastic leukemia (ALL) that express higher levels of HLA class I molecules than AML blasts. In addition, the presence of activating KIRs can also be assessed by analyzing the KIR genotype and using appropriate redirected killing assays (Chewning et al., 2007). Cytolytic activity of donor NK cells against patient’s leukemic blasts or, alternatively, against appropriate EBV-induced B cell lines should be evaluated to select the HSCT donor with the best alloreactive capacity. In general, the degree of cytolytic activity correlates with the size of phenotypically defined alloreactive NK cell subsets (Chewning et al., 2007; Pende et al., 2009).

The fact that alloreactive NK cells are generated in the recipient after the allograft was documented in the early studies by Ruggeri et al. (2002). More recent studies by our group have confirmed and extended these findings. Donor’s alloreactive NK cell populations have been identified on the basis of both phenotypic and functional (i.e., cytolytic activity) criteria in a large cohort of pediatric patients with high-risk leukemias even over 5 years after transplantation (Moretta et al., 2008, 2011). In these studies, a great variability in the size of the alloreactive NK cell population was detected in different donors and in post-transplantation patients. Importantly, most patients characterized by high proportions of alloreactive NK cells were disease-free after long time intervals (Pende et al., 2009). In addition, a correlation between the size of the alloreactive NK subset and the clinical outcome was found. After transplantation of positively selected CD34^+^ cells, KIR^+^ alloreactive NK cells were detectable at 6–7 weeks after transplantation and, in most instances, the pattern of expressed KIRs was similar to that originally found in the donor (Moretta et al., 2008, 2011; Pende et al., 2009).

A major and fascinating question is why alloreactive NK cells do not mediate GvHD. Early experimental evidence suggested that NK cells predominantly attack the hematopoietic cells of the host, while sparing tissues that are common targets of T cell-mediated GvHD. For example, in the hybrid resistance phenomenon in the mouse, NK cells rejected BM graft, but did not attack other tissues (Bennet, 1987). More recent studies in mice showed that allogeneic cells can mediate GvL effect in the absence of GvHD (Asai et al., 1998). Ruggeri et al. (2002) obtained direct evidence that murine alloreactive NK cells did not cause GvHD, whereas infusion of allogeneic T cells killed all the mice. In the same murine model, alloreactive NK cells were also shown to kill host antigen-presenting cells. This effect can contribute to reduce the risk of GvHD. The molecular basis of the resistance of recipient normal tissues other than the hematopoietic ones is the lack of ligands for activating NK receptors. These ligands become expressed or up-regulated by cells of different histotypes upon cell stress, viral infection, or tumor transformation (Moretta et al., 2006). Accordingly, NK cells cannot attack normal resting cells.

Notably, recent reports have proposed a novel approach for optimal donor selection based on the KIR genotype analysis. These studies provide evidence that the selection of donors with KIR B haplotypes was associated with significant improvements in both overall and relapse free survival, suggesting that activating KIRs, particularly those located in the centromeric portion, play a positive role in GvL in adult AML patients (Cooley et al., 2010; Symons et al., 2010).

It should be mentioned that some studies failed to establish an association between the presence of donor NK alloreactivity and a favorable clinical outcome of transplanted patients (Leung et al., 2004; Nguyen et al., 2005; Vago et al., 2008). This can be explained taking into account (1) the type of grafted cells (manipulated vs un-manipulated), (2) the type of conditioning regimen, (3) the source (PBSC vs BM) and, importantly, the number of stem cells used (“megadoses” in haplo-HSCT), (4) the type of GvHD prophylaxis, and (5) the clinical status of the patient at the time of the allograft (early vs advanced disease).

## RECENT ADVANCES AND FUTURE PERSPECTIVES

There is no doubt that studies on NK cell receptor specificity and function allowed a rapid exploitation of these results in the treatment of high risk leukemias. Nonetheless, further relevant progresses are expected from the use of donor alloreactive NK cells as a tool for improving the clinical outcome of severe malignancies and for preventing GvHD.

The capability of alloreactive NK cells to kill host DCs, which are known to initiate T cell-mediated GvHD through presentation of host alloantigens to donor T cells, suggested a novel and interesting experimental approach in mice (Asai et al., 1998; Shlomchik et al., 1999). Infusion of mature, donor-vs-recipient alloreactive NK cells prevented GvHD to such an extent that mice that were given these cells could receive mismatched BM grafts containing up to 30 times the lethal dose of allogeneic T cells in the absence of clinical or histological evidence of GvHD (Asai et al., 1998). Transfer of such an approach to humans is particularly promising to prevent or treat GvHD, in view of the role of the lytic activity of donor-derived NK cells toward recipient T lymphocytes in the control/prevention of graft rejection.

**FIGURE 1:**
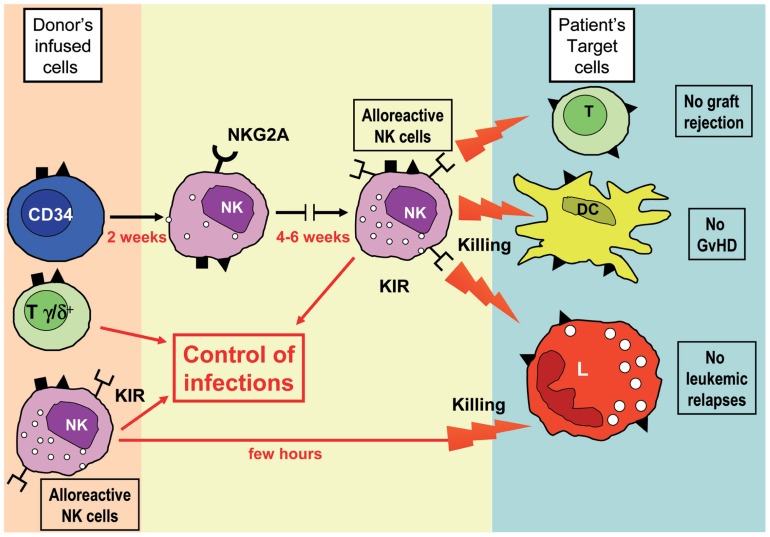
**A novel strategy for HSC transplantation from haploidentical donors**. In this protocol, HSC-enriched cell populations are obtained by negative selection upon removal of TCR α/β^+^ T cells and CD19^+^ B cells. Notably, in addition to CD34^+^ cells, these cell suspensions contain mature NK cells and TCR γ/δ^+^ T cells. Using this strategy, two sources of alloreactive NK cells will come into play: (1) those generated from CD34^+^ cells after 6–8 weeks from transplantation and (2) those present in the fresh cell suspension infused into patients. It is evident that the prompt availability of alloreactive effector cells may greatly improve the anti-leukemia effect and the removal of residual patient’s DCs and T lymphocytes, thus ensuring a more efficient prevention of leukemic relapses, GvHD and graft-rejection. In addition, transplanted NK and γ/δ T cells may provide a first line of defense against different infectious agents.

As mentioned above, in the haplo-HSCT setting, after the infusion of pure CD34^+^ cells, the first appearance of KIR^+^ alloreactive NK cells from HSC precursors may require 6–8 weeks and thus their anti-leukemia effect is relatively delayed. In case of high residual tumor burden and/or of rapidly proliferating leukemia blasts, this may result in leukemic relapses. To minimize this risk, mature alloreactive NK cells isolated from the haploidentical donor may be infused at short time intervals after HSCT. These mature donor NK cells could be properly activated *ex vivo* with interleukin-15 for further improving the clinical results of haplo-HSCT. Another promising and even less cumbersome approach is represented by the use of a recently developed method of graft manipulation based on the negative selection of T lymphocytes carrying the α/β chains of the T cell receptor (TCR) coupled with a B cell-depletion through an anti-CD19 mAb. T lymphocytes carrying the α/β chains of TCR are the lymphocyte subset responsible for the occurrence of GvHD, and thus their elimination allows to prevent the occurrence of this life-threatening complication of an allograft. This novel approach permits to transfer to the recipient not only high numbers of CD34^+^ cells, but also of mature donor NK cells and TCRγ/δ^+^ T cells which can display their protective effect against leukemia re-growth and life-threatening infections (Chaleff et al., 2007; Handgretinger, 2012). Alloreactive NK cells are immediately available and may promptly exert their anti-leukemic and GvHD-preventing effect (**Figure 1**). A formal clinical trial using this approach is ongoing in our department and the preliminary results are extremely encouraging (Locatelli et al., unpublished). Likewise, preliminary experimental data indicate that, already 1 month after the allograft, pediatric patients receiving this novel type of HSCT from an HLA-haploidentical donor have peripheral mature NK cells that fully express KIRs and are endowed with a good lytic capacity against leukemia cells.

Regarding other possible settings in which alloreactive NK cells can be of relevant clinical interest, recent studies reported on the infusion of third-party purified NK cells in patients with either relapsed or first CR AML, who had not received allogeneic HSCT (Miller et al., 2005; Rubnitz et al., 2010). These patients were given immunosuppressive chemotherapy (combining fludarabine and cyclophosphamide) and interleukin-2, respectively, before and after NK cell infusion in order to prevent rejection and favor NK cell function. NK cells transiently engrafted and expanded *in vivo*. The clinical results were particularly encouraging. This appears as a promising novel therapy for reducing the risk of relapse in patients with AML treated with conventional chemotherapy. Another promising approach to control leukemia progression resides in the NK cell manipulation using anti-KIR mAb (Romagné et al., 2009). This mAb, currently tested in phase II clinical trials on patients with AML or multiple myeloma, confers specific, stable blockade of KIR and induces NK-mediated killing of HLA-matched tumor cells *in vitro* and *in vivo*.

Altogether these data indicate that the discovery of NK receptors and NK cell alloreactivity has represented a true revolution in the field of allo-HSCT, underlining that not only adaptive immunity, but also innate immunity may be crucial for guaranteeing a successful clinical outcome.

## Conflict of Interest Statement

Alessandro Moretta is a founder and shareholder of Innate-Pharma (Marseille, France). The remaining authors declare no competing financial interests.
